# Quality Gaps in Online Media Coverage of Antiamyloid Monoclonal Antibodies for Alzheimer Disease

**DOI:** 10.1001/jamanetworkopen.2026.5026

**Published:** 2026-03-31

**Authors:** Arthur C. Macedo, Arthur Gobbi de Lima, Isabela Sheng Ling Miaw, Isabella Harb Bizzi, Jesus Mística Ventura Balbino, Joseph Therriault, Eduardo R. Zimmer, Pietro Ghezzi, Pedro Rosa-Neto

**Affiliations:** 1 Translational Neuroimaging Laboratory, The McGill University Research Centre for Studies in Aging, McConnell Brain Imaging Centre, Montreal Neurological Institute, Montréal, Québec, Canada; 2 Department of Neurology and Neurosurgery, McGill University, Montréal, Québec, Canada; 3 Montreal Neurological Institute, Montréal, Québec, Canada; 4 Hospital das Clínicas da Faculdade de Medicina da Universidade de São Paulo, São Paulo, Brazil; 5 Faculdade de Medicina, Universidade Federal de Minas Gerais, Minas Gerais, Brazil; 6 Faculdade São Leopoldo Mandic, Campinas, São Paulo, Brazil; 7 Hospital Moinhos de Vento, Porto Alegre, Brazil; 8 Department of Pharmacology, Universidade Federal do Rio Grande do Sul, Porto Alegre, Brazil; 9 Graduate Program of Biological Sciences: Biochemistry, Universidade Federal do Rio Grande do Sul, Porto Alegre, Brazil; 10 Graduate Program of Biological Sciences: Pharmacology and Therapeutics, Universidade Federal do Rio Grande do Sul, Porto Alegre, Brazil; 11 Department of Biomolecular Sciences, University of Urbino, Urbino, Italy; 12 Brighton and Sussex Medical School, Brighton, United Kingdom; 13 The Peter O’Donnell Jr Brain Institute, University of Texas Southwestern Medical Centre, Dallas

## Abstract

This cross-sectional study examines the quality of information on Alzheimer disease (AD) monoclonal antibodies presented by news websites.

## Introduction

The recently Food and Drug Administration (FDA)–approved anti–amyloid-β monoclonal antibodies (mAbs) represent a paradigm shift in Alzheimer disease (AD) management^1^ and have attracted substantial media attention. Although the media is a critical bridge between science and the public, it often conveys information that is not evidence based or accurate.^2,3^ Here, we evaluate the quality of information on AD mAbs presented by news websites.

## Methods

For this cross-sectional study, we searched the US–National collection of MediaCloud on January 29, 2023, using the terms *Alzheimer AND (therapy OR cure)* to identify records published since January 29, 2020. We then selected articles with *Alzheimer* in the title. Two independent assessors (I.S.L.M. and I.H.B.) screened records for mAb mentions; records without mAbs or inaccessible records were excluded. Assessors categorized each article’s stance toward mAbs as positive, negative, or neutral^2,3^ and whether articles mentioned FDA approval, biomarker testing requirements, and eligibility criteria for treatment. The quality of information reporting was evaluated by 2 other independent assessors (A.G.L. and J.M.V.B.) using the HealthNewsReview (HNR) criteria,^4^ an evidence-based framework for medical news assessment (score range, 0-10, with higher values indicating better reporting quality) . This study follows the STROBE reporting guidelines. This study did not require institutional review board approval under 45 CFR §46 because it did not involve human participants or identifiable private information. Statistical analyses were conducted in R version 4.4.1 (R Project for Statistical Computing). Statistical significance was determined by the Kruskal-Wallis or Mann-Whitney *U* test and was set at 2-sided *P* < .05. Additional details are shown in the eMethods and eTable in Supplement 1.

## Results

The search yielded 1300 records; 342 were selected following title filtering. Among these, 189 mentioned mAbs, and 27 were inaccessible because of broken links or paywalls. Specifically, 147 articles cited lecanemab, 128 aducanumab, and 27 donanemab.

**Figure 1.**
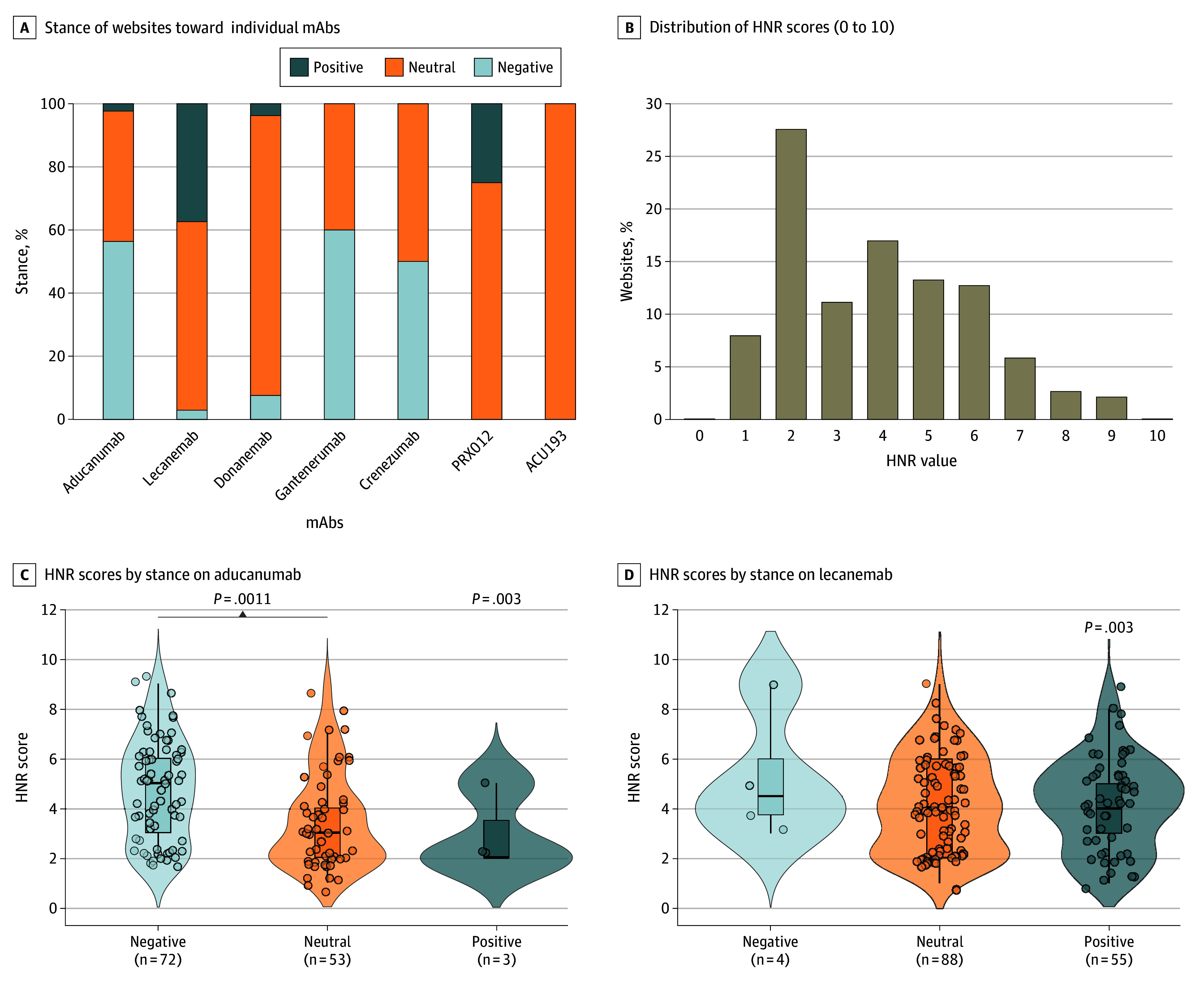
Bar Graph of Article Stance Toward Antiamyloid Monoclonal Antibodies (mAbs), Bar Graph of HealthNewsReview (HNR) Scores, and Violin Plots of HNR Score Distributions A, Stacked bar chart displays the stance of websites toward individual mAbs, categorized as positive, neutral, or negative. B, Bar graph shows the distribution of HNR scores (range, 0-10) across all included news articles. C and D, Violin plots show the distribution of HNR scores stratified by article stance toward aducanumab (C) and lecanemab (D), which were selected for comparative analyses as the 2 most frequently mentioned mAbs. Violin plots depict score distributions, with overlaid points representing individual articles; boxes indicate medians and IQRs. Whiskers extend to the most extreme observed values within 1.5 times the IQR from the first and third quartiles. Groupwise differences were assessed using the Kruskal-Wallis test (*P* values on the top right), with pairwise comparisons performed using the Mann-Whitney *U* test.

Most websites citing aducanumab (72 websites [56%]) expressed a negative stance, whereas negative coverage was uncommon for lecanemab (4 websites [3%]) (Figure 1A). Notably, 149 websites (79%) reported FDA approval status, but fewer noted eligibility restrictions (89 websites [47%]) or the need for biomarker testing before initiating treatment (51 websites [27%]).

**Figure 2.**
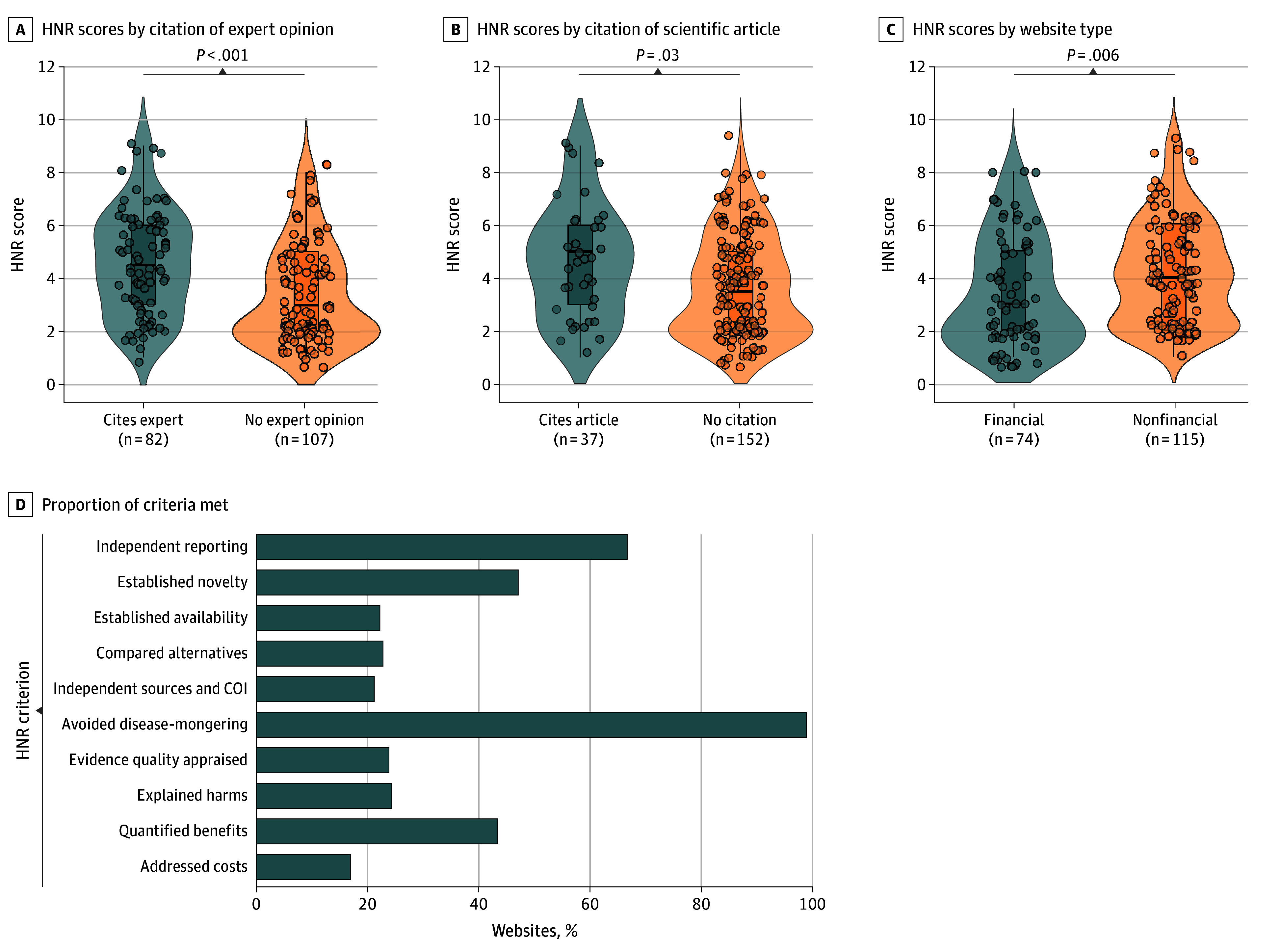
Violin Plots of Differences in HealthNewsReview (HNR) Scores Across Article Characteristics and Bar Graph of Adherence to Individual HNR Criteria A-C, Violin plots show distributions of HNR scores, with individual articles shown as points and boxes indicating medians and IQRs. Whiskers extend to the most extreme observed values within 1.5 times the IQR from the first and third quartiles. Panel A compares articles that cited expert opinion with those that did not. Panel B compares articles that cited a scientific publication with those that included no citation. Panel C compares articles published by financial vs nonfinancial outlets. Between-group differences were assessed using the Mann-Whitney *U* test. D, Bar graph shows the proportion of articles meeting each HNR reporting criterion. COI indicates conflicts of interest.

The median (IQR) HNR score was 4 (2-5) (Figure 1B). HNR scores differed by aducanumab stance, with higher scores among negative vs neutral articles (*r_rb_* = 0.34; *P* = .001) (Figure 1C-D). HNR scores were higher for nonfinancial vs financial outlets (*r_rb_* = 0.24; *P* = .006), and for articles including expert opinion (*r_rb_* = 0.36; *P* < .001) or linking to scientific publications (*r_rb_* = 0.23; *P* = .03) (Figure 2A-C). The lowest fulfillment rates were for criteria addressing costs (32 websites [17%]), conflicts of interest or independent sources (40 websites [21%]), and availability of the therapy (42 websites [22%]). Potential harms were discussed in 46 articles (24%) (Figure 2D).

## Discussion

In this cross-sectional study, we evaluated the content and quality of information provided by news retrieved through an online search on AD therapies. We found that most basic standards for high-quality health information were not met by most websites.

Our findings highlight the prominent attention that mAbs have received in media coverage, although the media stance varied across therapies. Aducanumab was predominantly portrayed negatively, whereas lecanemab was more often described positively, likely reflecting differences in available clinical evidence and regulatory decisions at the time of analysis.^1,5^

Few articles cited experts or scientific sources; however, those that did had higher HNR scores. Key information was frequently omitted, including eligibility criteria and the requirement for biomarker confirmation. Omitting biomarker requirements obscures additional testing burdens and costs that may further limit effective treatment eligibility.^6^ Correspondingly, costs were the poorest-performing HNR criterion. Despite the known frequency and potential severity of adverse effects associated with mAbs,^1^ potential harms were discussed in only 24% of articles.

Limitations should be considered. Our sample was restricted to US-based websites and to articles accessible without paywalls, limiting generalizability. In addition, the search strategy prioritized a simple and reproducible approach and did not include terms related to mild cognitive impairment or other treatment-related keywords, potentially leading to underrepresentation of relevant media content. In conclusion, media coverage of AD mAbs is limited in content and completeness, highlighting the need to improve standards of health news reporting to prevent unrealistic expectations among the public.

## Notes

Supplement 1. eMethods. eReferences eTable. Minimum requirements to satisfy the HealthNewsReview criteria

Supplement 2. Data Sharing Statement
