# Supplementary material for: Quality Gaps in Online Media Coverage of Antiamyloid Monoclonal Antibodies for Alzheimer Disease
Source: JAMA Netw Open. 2026 Mar 31;9(3):e265026. doi: 10.1001/jamanetworkopen.2026.5026 (PMC13040397; doi:10.1001/jamanetworkopen.2026.5026)
Supplement: Supplement 1. — eMethods. eReferences eTable. Minimum requirements to satisfy the HealthNewsReview criteria [file jamanetwopen-e265026-s001.pdf]

Supplemental Online Content

Macedo AC, de Lima AG, Miaw ISL, et al. Quality gaps in online media coverage of antiamyloid monoclonal antibodies for Alzheimer disease. *JAMA Netw Open*. 2026;9(4):e265026. doi:10.1001/jamanetworkopen.2026.5026

eMethods
eReferences
eTable. Minimum requirements to satisfy the HealthNewsReview criteria

This supplemental material has been provided by the authors to give readers additional information about their work.

© 2026 Macedo AC et al. *JAMA Network Open*.

eMethods

Search Strategy

In this cross-sectional study of media content, we searched the collection "US-National" of the database MediaCloud (www.mediacloud.org) on January 29, 2023, using the terms "Alzheimer* AND (therapy OR cure)". The search strategy used a limited set of keywords likely to be used by patients seeking news on Alzheimer disease (AD) therapies. Unlike a systematic review, the objective was not exhaustive retrieval of all media content, but rather the use of a simple and reproducible approach to identify a manageable and representative sample of news articles for consistent screening and systematic evaluation of reporting quality.

The United States was selected as the study setting to focus on media coverage in the country where anti-amyloid monoclonal antibodies (mAbs) were first approved by a regulatory agency. Articles published between January 29, 2020, and January 29, 2023, were included to capture news released after the US Food and Drug Administration (FDA) approval of the first mAb for AD, aducanumab. Following the initial search, articles were included if the word "Alzheimer" appeared in the title.

Content Assessment

Two independent assessors systematically reviewed the selected articles to extract relevant data. The first step was to select articles mentioning mAbs. Those not mentioning mAbs or not accessible due to paywalls or broken links were excluded.

From included articles, assessors recorded the specific mAbs mentioned (e.g., aducanumab, lecanemab, donanemab). Moreover, assessors categorized the stance

© 2026 Macedo AC et al. *JAMA Network Open*.

of the article regarding each therapy as positive, negative, or neutral, as done in previous research[1,2]. They also examined whether the articles mentioned FDA approval, the requirement for biomarker testing prior to initiating therapy, and the eligibility criteria for treatment (e.g., cognitive impairment level)[3,4].

To assess completeness, the presence of references to scientific publications was evaluated, as this is a key criterion for quality journalism in healthcare reporting[5]. Articles were also reviewed to determine whether they included expert opinions, here defined as individuals with recognized academic or professional authority in the field. All data were collected independently, and disagreements were resolved by consulting a third assessor.

Of note, we recorded whether each news outlet was financial/business-related, since typology of websites has been shown to affect reporting quality and transparency[1,2]. Outlets identified as such included benzinga.com, cnbc.com, forbes.com, foxbusiness.com, ibtimes.com, investors.com, thestreet.com, and wsj.com.

Quality of Reporting Assessment

The quality of the information reporting was evaluated using the HealthNewsReview (HNR) criteria, an evidence-based framework used to assess the quality of medical reporting[5,6]. Two neurologists underwent training before independently scoring the articles. The criteria comprise 10 key elements that address whether the news provides sufficient information for consumers to make informed decisions about medical interventions. These elements include coverage of benefits, harms, costs, conflicts of interest, and comparisons to existing treatments.

The specific criteria and their thresholds for a "sufficient" rating are presented in the **eTable**. Disagreements between the raters were resolved through consultation with a third assessor. The methodology aligns with previous studies that have utilized the HNR framework for evaluating medical journalism[7].

Statistical analyses

Statistical analyses were conducted in R version 4.4.1. For groupwise comparisons of continuous variables (HNR scores) across more than two groups, we applied the Kruskal-Wallis test, requiring at least two observations per group. When each group contained at least five observations, pairwise comparisons were performed using the Mann-Whitney U test. Effect sizes for pairwise comparisons were quantified using the rank-biserial correlation (r_{rb}).

eReferences

1. Macedo AC, de Faria AOV, Bizzi I, Moreira FA, Colasanti A, Ghezzi P. Online information on medical cannabis is not always aligned with scientific evidence and may raise unrealistic expectations. *J Cannabis Res*. 2022;4(1):37.

2. Macedo AC, de Faria AOV, Ghezzi P. Boosting the Immune System, From Science to Myth: Analysis the Infosphere With Google. *Front Med (Lausanne)*. 2019;6:165.

3. Cummings J, Apostolova L, Rabinovici GD, et al. Lecanemab: Appropriate Use Recommendations. *J Prev Alzheimers Dis*. 2023;10(3):362-377.

4. Rabinovici GD, Selkoe DJ, Schindler SE, et al. Donanemab: Appropriate use recommendations. *J Prev Alzheimers Dis*. 2025;12(5):100150.

© 2026 Macedo AC et al. *JAMA Network Open*.

5. Schwitzer G. How do US journalists cover treatments, tests, products, and procedures? An evaluation of 500 stories. *PLoS Med*. 2008;5(5):e95.

6. Schwitzer G. A Guide to Reading Health Care News Stories. *JAMA Intern Med.* 2014;174(7):1183–1186.

7. Al-Jefri M, Evans R, Lee J, Ghezzi P. Automatic Identification of Information Quality Metrics in Health News Stories. *Front Public Health*. 2020;8:515347.

© 2026 Macedo AC et al. *JAMA Network Open*.

eTable. Minimum requirements to satisfy the HealthNewsReview criteria

Criterion	Minimum requirements (1 = satisfied, 0 = not satisfied)
**#1 - Does the story adequately discuss the costs of the intervention?**	• Mentions the cost of the medication (quantified or estimated, and/or compared with existing therapies)<br>• Mentions costs of required or commonly associated procedures (e.g., specialist consultations, biomarker or genetic testing, infusion administration, monitoring visits), even if not quantified
**#2 - Does the story adequately quantify the benefits of the intervention?**	• Quantifies benefits with numerical terms (e.g., “30% reduction in cognitive decline”), rather than with vague descriptors (“mild,” “moderate,” “important”)<br>• Specifies the nature of the benefit (e.g., “slowed memory decline over 18 months”) instead of using general statements (“improved patient health”)
**#3 - Does the story adequately explain/quantify the harms of the intervention?**	• Describes both the frequency and severity of side effects<br>• Addresses both major (e.g., ARIA) and minor side effects
**#4 - Does the story seem to grasp the quality of the evidence?**	• States whether the evidence comes from randomized controlled trials (RCTs) or other study types<br>• Discusses key limitations (e.g., uncertainty of clinical benefit for aducanumab; limited follow-up of RCTs; unanswered questions such as treatment duration or persistence of benefit after stopping)
**#5 - Does the story commit disease-mongering?**	• Does not exaggerate Alzheimer’s disease prevalence<br>• Does not misrepresent AD (e.g., equating normal memory loss with AD)
**#6 - Does the story use independent sources and identify conflicts of interest?**	• Reports relevant conflicts of interest (e.g., pharmaceutical company involvement)<br>• Identifies the source of information (e.g., news release, journal article, editorial, conference presentation, input from an expert or trusted source)
**#7 - Does the story compare the new approach with existing alternatives?**	• Discusses advantages (e.g., disease-modifying potential) and disadvantages (e.g., no evidence of symptom improvement for neuropsychiatric symptoms) of the mAb compared with existing options<br>• Explains how the new therapy fits within existing treatment approaches (e.g., mAb may need to be combined with symptomatic drugs)
**#8 - Does the story establish the availability of the treatment/test/product/procedure?**	• States whether and when the drug is available (e.g., pharmacy access, prescribable by doctors at the time of publication)<br>• If pre-FDA approval, clarifies that it is not yet available and outlines remaining regulatory steps
**#9 - Does the story establish the true novelty of the approach?**	• Explains what makes mAbs new or different (e.g., targets AD pathology, has disease-modifying potential)<br>• Distinguishes genuine novelty from hype (e.g., avoids vague terms like “groundbreaking” without a clear explanation)
**#10 - Does the story appear to rely solely or largely on a news release?**	• Provides information beyond a company or institutional press release (i.e., not solely or largely based on it)

AD: Alzheimer’s disease; ARIA: amyloid-related imaging abnormalities; mAb: monoclonal antibodies; FDA: Food and Drug Administration; RCT: randomized controlled trial.

© 2026 Macedo AC et al. *JAMA Network Open*.
