# Supplementary material for: Quality Gaps in Online Media Coverage of Antiamyloid Monoclonal Antibodies for Alzheimer Disease
Source: JAMA Netw Open. 2026 Mar 31;9(3):e265026. doi: 10.1001/jamanetworkopen.2026.5026 (PMC13040397; doi:10.1001/jamanetworkopen.2026.5026)
Supplement: Supplement 2. — Data Sharing Statement [file jamanetwopen-e265026-s002.pdf]

Data Sharing Statement

Macedo. Quality Gaps in Online Media Coverage of Antiamyloid Monoclonal Antibodies for Alzheimer Disease. *JAMA Netw Open*. Published April 03, 2026. doi:10.1001/jamanetworkopen.2026.5026

Data
Data available: Yes
Data types: Data (not involving human participants)
How to access data: The data underlying this study consist of variables derived from the authors' systematic assessment of publicly available online news articles. The analytic dataset will be made available upon reasonable request to the corresponding author for purposes of reproducing the results reported in this article. Requests will be evaluated on a case-by-case basis.
When available: With publication

Supporting Documents
Document types: None

Additional Information
Who can access the data: The data underlying this study consist of variables derived from the authors' systematic assessment of publicly available online news articles. The analytic dataset will be made available upon reasonable request to the corresponding author for purposes of reproducing the results reported in this article. Requests will be evaluated on a case-by-case basis.
Types of analyses: The data underlying this study consist of variables derived from the authors' systematic assessment of publicly available online news articles. The analytic dataset will be made available upon reasonable request to the corresponding author for purposes of reproducing the results reported in this article. Requests will be evaluated on a case-by-case basis.
Mechanisms of data availability: The data underlying this study consist of variables derived from the authors' systematic assessment of publicly available online news articles. The analytic dataset will be made available upon reasonable request to the corresponding author for purposes of reproducing the results reported in this article. Requests will be evaluated on a case-by-case basis.
